# Enhanced differentiation of functional human T cells in NSGW41 mice with tissue-specific expression of human interleukin-7

**DOI:** 10.1038/s41375-021-01259-5

**Published:** 2021-05-11

**Authors:** Emilie Coppin, Bala Sai Sundarasetty, Susann Rahmig, Jonas Blume, Nikita A. Verheyden, Franz Bahlmann, Sarina Ravens, Undine Schubert, Janine Schmid, Stefan Ludwig, Katharina Geissler, Orlando Guntinas-Lichius, Constantin von Kaisenberg, Tanja Groten, Alexander Platz, Ronald Naumann, Barbara Ludwig, Immo Prinz, Claudia Waskow, Andreas Krueger

**Affiliations:** 1 Regeneration in Hematopoiesis, Institute for Immunology, TU Dresden, Dresden, Germany; 2 Immunology of Aging, Leibniz Institute on Aging—Fritz Lipmann Institute, Jena, Germany; 3 Institute for Molecular Medicine, Goethe University Frankfurt am Main, Frankfurt am Main, Germany; 4 Institute of Immunology, Hannover Medical School, Hannover, Germany; 5 Department of Obstetrics and Gynecology, Bürgerhospital Frankfurt, Frankfurt am Main, Germany; 6 Department of Medicine III, Technical University Dresden, Dresden, Germany; 7 German Center for Diabetes Research (DZD e.V.), Neuherberg, Germany; 8 Department of Visceral-, Thorax- and Vascular Surgery, Technical University Dresden, Dresden, Germany; 9 Department of Otorhinolaryngology, Jena University Hospital, Jena, Germany; 10 Department Obstetrics, Gynecology and Reproductive Medicine, Hannover Medical School, Hannover, Germany; 11 Department of Obstetrics, Jena University Hospital, Jena, Germany; 12 DKMS Cord Blood Bank, Dresden, Germany; 13 Max-Planck Institute of Molecular Cell Biology and Genetics, Dresden, Germany; 14 Faculty of Biological Sciences, Institute of Biochemistry and Biophysics, Friedrich-Schiller-University Jena, Jena, Germany; 15 Present Address: Recombinant Technologies, CSL Behring GmbH, Marburg, Germany

## Abstract

Humanized mouse models have become increasingly valuable tools to study human hematopoiesis and infectious diseases. However, human T-cell differentiation remains inefficient. We generated mice expressing human interleukin-7 (IL-7), a critical growth and survival factor for T cells, under the control of murine IL-7 regulatory elements. After transfer of human cord blood-derived hematopoietic stem and progenitor cells, transgenic mice on the NSGW41 background, termed NSGW41hIL7, showed elevated and prolonged human cellularity in the thymus while maintaining physiological ratios of thymocyte subsets. As a consequence, numbers of functional human T cells in the periphery were increased without evidence for pathological lymphoproliferation or aberrant expansion of effector or memory-like T cells. We conclude that the novel NSGW41hIL7 strain represents an optimized mouse model for humanization to better understand human T-cell differentiation in vivo and to generate a human immune system with a better approximation of human lymphocyte ratios.

## Introduction

Humanized mouse models have emerged as indispensable tools for improving our understanding of human hematopoiesis and the human immune system. However, efficient differentiation of human T cells remains a challenge in humanized mice and we have focused on interleukin-7 (IL-7) as a key factor for lymphocyte survival and proliferation to improve that situation [1–4]. In vitro, human (h)IL-7 was 100-fold more potent to expand and differentiate human T-cell progenitors when compared to murine (m)IL-7 [4]. However, unrestricted supply of IL-7 results in the generation of lymphomas in mice [5]. Further, excessive amounts of mIL-7 limit T-cell differentiation by interfering with Notch signaling [6, 7]. In fact, administration of recombinant hIL-7 to humanized mice unfavorably shifted the balance between peripheral T and B cells and displayed only a transient benefit in the thymus [4, 8]. Furthermore, lentivirus-based ectopic expression of hIL-7 by human donor cells did not improve T-cell differentiation in humanized mice [4]. We, therefore, hypothesized that dose and spatially restricted availability of hIL-7 might be essential to improve human T-cell differentiation in humanized mice while simultaneously avoiding unwanted effects caused by excessive and spatially unrestricted availability of hIL-7. To this end, we generated hIL-7 bacterial artificial chromosome (BAC) transgenic NSGW41 mice as a tool to study human T-cell differentiation in vivo.

## Materials and methods

A BAC containing codon-optimized cDNA of human *IL7* (corresponding to protein NP_000871) introduced at the 3′ end of the 5′UTR of the *Il7* gene flanked by 96 kb upstream, and the entire *Il7* locus plus an additional 17 kb downstream was constructed according to a described strategy and used to generate NODhIL7 mice directly using the NOD genetic background [9]. Offspring showing detectable expression of hIL-7 mRNA was crossed with NSGW41 mice. All animal experiments were performed in accordance with German animal welfare legislation and were approved by the relevant authorities: Landesdirektion Dresden, the Thüringer Landesamt für Verbraucherschutz (TLV), the Niedersächsisches Landesamt für Verbraucherschutz und Lebensmittelsicherheit (LAVES), and the Regierungspräsidium Darmstadt. Further materials and methods can be found in the supplemental material and Table S1.

## Results and discussion

**Fig. 1:**
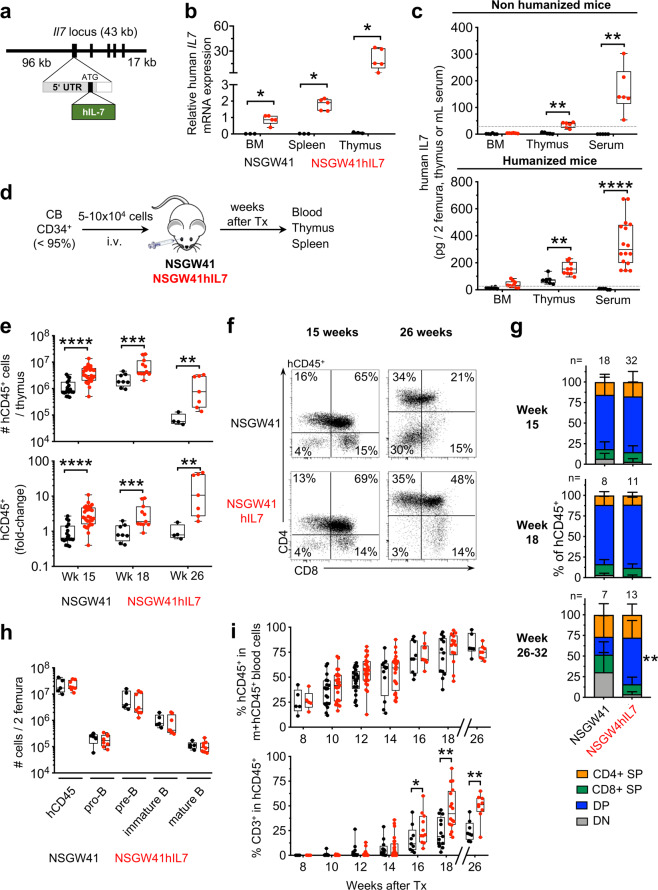
Improved intrathymic human T cell differentiation in NSGW41hIL7 mice. **a** Scheme of BAC constructs for the generation of NSGW41hIL7 mice. **b** Abundance of hIL-7 transcript in bone marrow (BM), spleen, and thymus from humanized NSGW41 or NSGW41hIL7 mice. **c** hIL-7 protein levels in bone marrow, thymus, and serum isolated from non-humanized NSGW41 (black) or NSGW41hIL7 mice (red, top) and from NSGW41 or NSGW41hIL7 mice that have received human CD34^+^ HSPCs 26-38 weeks before (bottom). Gray lines indicate the limit of assay sensitivity. **d** Scheme of transplantation experiments. **e** Numbers (top) and fold-change (bottom) of human (h)CD45^+^ cells in thymi of humanized NSGW41 or NSGW41hIL7 mice at the indicated time points after humanization. Fold-changes were calculated by dividing hCD45^+^ thymocyte numbers from humanized NSGW41hIL7 mice by the thymocyte numbers from humanized NSGW41 mice. This was conducted separately for each experiment and the results pooled. **f** Analysis of CD4 and CD8 expression on hCD45^+^ thymocytes from NSGW41 or NSGW41hIL7 mice that have received human HPSCs 15 (left) or 26 (right) weeks before. **g** Composition of thymocyte subsets in NSGW41 or NSGW41hIL7 mice at the indicated time points after humanization. Frequencies of DN populations were significantly decreased in NSGW41hIL7 compared to NSGW41 counterparts (week 15 and 18: *P* < 0.01 and week 32: *P* < 0.001). **h** Numbers of B cell subsets in the bone marrow of NSGW41 or NSGW41hIL7 mice 26–32 weeks after humanization. **i** Kinetics of the appearance of human CD45^+^ cells (hCD45^+^, top) and hCD3^+^ T cells within human leukocytes (bottom) in the blood after humanization. **b**, **c**, **e**, **h**, **i** Each dot represents an individual mouse. Boxes and whiskers indicate quartiles and median.

To generate a mouse model with tissue-specific expression of human (h)IL-7, we inserted cDNA encoding *IL7* into a BAC containing regulatory elements of the murine *Il7* locus, which has previously been demonstrated to faithfully direct expression of a reporter gene for mIL-7 expression (Fig. 1a) [9]. Transgenic mice were crossed to the NSGW41 strain, which carries the hypomorph W41 allele in the *Kit* gene, harbors the NOD-specific variant of the *Sirpa* gene, is T-, B- and NK-cell deficient based on null mutations in *Prkdc* and *Il2rg* genes, respectively, and allows for human donor stem cell engraftment in the absence of preconditioning, and were termed NSGW41hIL7 [10, 11]. NSGW41hIL7 mice contain three copies of the BAC transgene and expressed hIL-7 mRNA and protein in BM, spleen, and thymus (Fig. 1b, c). Upon transplantation of human CD34^+^-enriched cord blood cells into unconditioned NSGW41 or NSGW41hIL7 mice, human CD45^+^ cell numbers were 3.3-fold, 3.5-fold, and 21.2-fold higher in thymi from NSGW41hIL7 mice at 15, 18, and 26 weeks after reconstitution, respectively (Fig. 1d, e). Ratios of human CD4/CD8 double-negative (DN), double-positive (DP), and CD4 and CD8 single-positive (SP) thymocytes were comparable in both recipient lines 15 and 18 weeks after transplantation, indicating that expression of hIL-7 promotes bona fide T-cell differentiation (Fig. 1f, g). NSGW41hIL7 but not NSGW41 thymi predominantly contained DP thymocytes 26–32 weeks after transplantation, suggesting that hIL-7 supports human T-cell differentiation for extended periods of time in NSGW41hIL7 mice. Interestingly, this is contrary to a recently characterized combined knock-in of hIL-7 and hIL-15 on the NSG background that displayed massive skewing towards CD8 SP cells at the expense of DP thymocytes, possibly due to expression of hIL-15 [12]. In the periphery, these mice displayed NK-cell frequencies much larger than in human peripheral blood at the expense of other lymphocytes, possibly limiting their use to study T-cell responses. Consistent with the T-lineage specific role of IL-7 in human hematopoiesis, we observed no alterations in B-cell differentiation in NSGW41hIL7 mice compared to NSGW41 (Figs. 1h and S1a, b). We conclude that NSGW41hIL7 mice display improved and extended intrathymic T-cell differentiation from human cord blood-derived HSPCs.

Blood from NSGW41 or NSGW41hIL7 mice contained comparable levels of human hematopoietic cells peaking at 16–18 weeks after transplantation (Fig. 1i). Beginning at week 16 after transplantation frequencies of T cells among human CD45^+^ cells were significantly increased in NSGW41hIL7 mice compared to NSGW41 recipients, temporally coinciding with improved intrathymic T-cell differentiation.

**Fig. 2:**
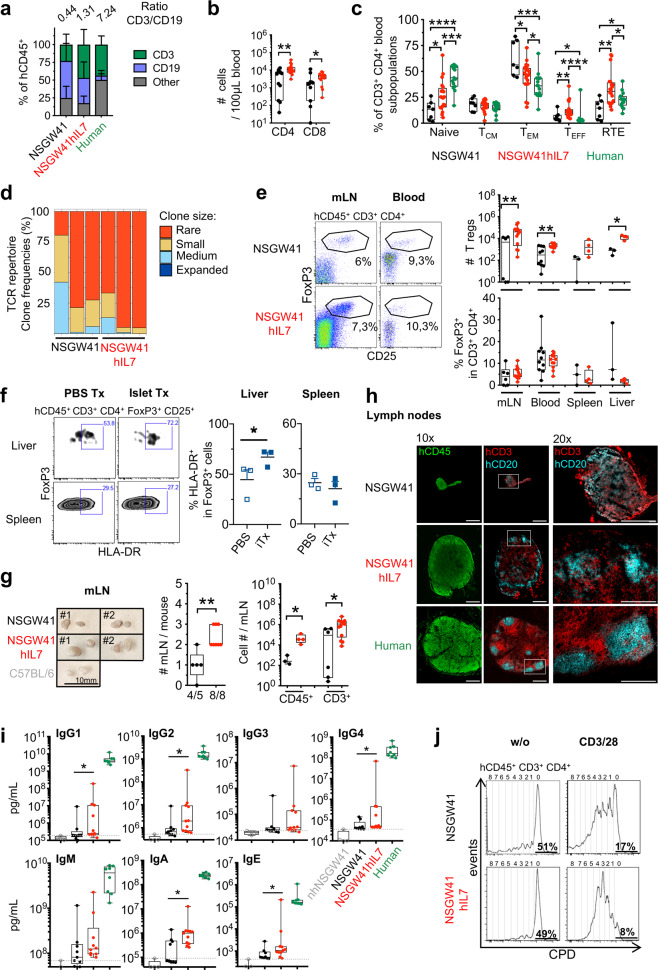
hIL-7-BAC transgene increases numbers of functional peripheral T cells in the absence of excessive lymphoproliferation. **a** Frequencies of T cells, B cells, and non-defined other cells of human origin in the blood of NSGW41 (*n* = 13), NSGW41hIL7 (*n* = 16) mice 18 weeks after humanization and human controls. Numbers on top indicate T vs. B cell ratios. **b** Human CD4^+^ and CD8^+^ T cells in the blood of humanized NSGW41 or NSGW41hIL7 mice 26 weeks after humanization. **c** Composition of blood CD4^+^ T cell subpopulations 26 weeks after humanization: Naïve T cells, central memory (*T*_*CM*_), effector memory (*T*_*EM*_), T effector (*T*_*EFF*_), and recent thymic emigrants (RTE) in NSGW41, NSGW41hIL7 mice, or human blood. **d** T cell receptor (TCR) repertoire diversity in splenic αβ T cells in NSGW41 or NSGW41hIL7 mice. Clones were binned into rare (0 < *X* ≤ 0.001), small (0.001 < *X* ≤ 0.01), medium (0.01 < *X* ≤ 0.1), and expanded (0.1 < *X* ≤ 1) (*n* = 3 per group). **e** Human CD4^+^ FoxP3^+^ Treg cells in humanized mice. Representative dot plots analyzing mesenteric lymph nodes (mLN) and blood (left) and numbers (right, top) and frequencies (right, bottom) of Tregs in mLN, blood, spleen, and liver of humanized NSGW41 or NSGW41hIL7 mice. **f** Human activated Tregs after intraportal xenotransplantation of porcine pancreatic islets into NSGW41hIL7 mice 26 weeks after humanization. Representative dot plots of liver and spleen analysis (left). Frequencies of HLA-DR^+^ FoxP3^+^ T cells in spleen and liver of humanized NSGW41hIL7 mice (right) 18 h after transplantation of islets (iTx) or PBS. **g** Photographs of mLN from humanized NSGW41 or NSGW41hIL7 mice isolated 26 weeks after humanization, or C57BL/6 controls (left). Quantity of mLNs per mouse (middle). Human CD45^+^ and hCD3^+^ cell numbers in mLNs from NSGW41 or NSGW41hIL7 mice (right). **h** Immunofluorescent images from lymph nodes from humanized NSGW41 or NSGW41hIL7 mice that were humanized 30 weeks before and a human cervical lymph node stained for human CD45 (green, left), CD3 (red), and CD20 (turquoise, middle and right). Areas of blow-up pictures (20× magnification) correspond to the white squares indicated in the 10× magnification images. Scale-bar: 500 µm. Data are representative of the analysis of lymph nodes from three mice per group. **i** Concentration of serum immunoglobulins in humanized mice. Levels of IgG1, IgG2, IgG3, IgG4, IgM, IgA, and IgE were determined from non-humanized (nh)NSGW41, humanized NSGW41, or humanized NSGW41hIL7 mice at 24–32 weeks after humanization. Human serum was used as positive control. Gray lines indicate the limit of assay sensitivity. **j** Activation of human T cells from NSGW41 (top) or NSGW41hIL7 mice (bottom). Histograms depict division of CPD-labeled spleen hCD3^+^ T cells 6 days after stimulation with CD3/28 beads (right) or control (w/o, left). Frequencies of non-divided human T cells and T cells that have divided 4, 5, or 6 times 6 days after stimulation. **b**, **c**, **e**, **f**, **g**, **i** Each dot represents an individual mouse.

Ratios of T and B cells increased progressively over time, with T cells ultimately becoming the predominant lymphocyte population in the blood of NSGW41hIL7 mice (Fig. 2a). T/B ratios >1 are also observed in human blood. Consistently, in spleens, human CD45^+^ leukocytes were increased in NSGW41hIL7 mice compared to NSGW41 mice, which was mainly attributable to increased numbers of T cells (Fig. S2a, b). We have shown before that human myelopoiesis, megakaryopoiesis, and erythropoiesis are significantly improved in humanized NSGW41 compared to NSG recipient mice [10, 13, 14]. Consistently, numbers of NK cells, myeloid cells, including monocytes, granulocytes, and dendritic cells (DCs) as well as platelets were largely comparable between NSGW41hIL7 mice and NSGW41 controls (Fig. S3a, b). Minor differences were observed in granulocytes in the liver, conventional DC, and plasmacytoid DC in bone marrow and liver, respectively. The underlying mechanisms remain to be elucidated.

We further characterized peripheral T cells to assess whether their numerical increase in NSGW41hIL7 mice reflected increased thymic output rather than hIL-7-induced peripheral expansion. CD4^+^ and CD8^+^ T cell numbers in blood were increased to a comparable extent (Fig. 2b). Within CD4^+^ T cells, frequencies of naive T cells and recent thymic emigrants (RTEs) and effector T cells increased in NSGW41hIL7 mice compared to NSGW41 (Figs. 2c and S4a, b). In contrast, frequencies of effector memory cells were reduced. In CD8^+^ T cells, a similar increase in naive and RTE and decrease in effector memory frequencies was observed, whereas frequencies of T effector and central memory subsets remained comparable to those detected in NSGW41 mice. Together, the relative contribution of different subsets to human T cells in NSGW41hIL7 mice resembled human peripheral blood. Analysis of T-cell receptor (TCR) repertoire diversity revealed a high frequency of rare T-cell clones, which were comparable between NSGW41 and NSGW41hIL7 mice. Virtually no expanded clones were observed, indicating the absence of hIL-7-induced lymphoproliferation (Fig. 2d). Furthermore, we observed similar frequencies of regulatory T (Treg) cells in multiple organs from NSGW41hIL7 or NSGW41 mice and therefore increased absolute numbers in NSGW41hIL7 mice, making their analysis feasible (Fig. 2e). To study the activation of Treg cells in humanized NSGW41hIL7 mice, we transplanted porcine pancreatic islets into the portal vein of humanized NSGW41hIL7 mice [13]. Eighteen hours after xenotransplantation, Treg cells in the liver but not the spleen displayed significantly increased expression of HLA-DR evidencing site-specific activation through the xenograft (Fig. 2f). Conventional T cells remained largely unaltered in peripheral lymphoid organs but showed modest signs of activation in the liver after islet transplantation (Fig. S5a, b). Further, mesenteric lymph nodes (mLNs) were increased in number and individual size in NSGW41hIL7 mice compared to NSGW41, suggesting that NSGW41hIL7 mice constitute an improved model for studying gut-associated immune responses (Fig. 2g). Immunofluorescence analysis revealed confined B-cell areas in mLNs of NSGW41hIL7 mice (Fig. 2h). In contrast, no overt structural organization of B cells was observed in mLNs from NSGW41 mice. These findings were reminiscent of humanized BRGST mice, which ectopically express murine TSLP [15]. However, humanized BRGST mice display a human T-cell compartment with expanded memory populations at the expense of naïve cells. Serum levels of IgG1, IgG2, IgG4, IgA, and IgE were significantly increased in unchallenged NSGW41hIL7 mice when compared to NSGW41 controls (Fig. 2i). To test for functionality, CD4^+^ T cells from NSGW41 or NSGW41hIL7 spleens were activated in vitro and displayed similar levels of activation visualized by the expression of CD25 and CD69 (Fig. S6a). Finally, NSGW41hIL7-derived CD4^+^ and CD8^+^ T cells displayed increased division in response to anti-CD3/CD28 or phytohemagglutinin stimulation (Figs. 2j and S6b, c). We conclude that human T cells differentiated in NSGW41hIL7 mice respond efficiently to TCR triggering in vivo and ex vivo.

Taken together, this study adds another critical component to the toolbox available for progressive optimization of mouse models with a human immune system taking advantage of naturally occurring mutations and genetic engineering, also allowing for analysis of rare T cell subsets, including T-cell progenitors as well as Treg cells. In addition, a full complement of T-cell subsets may also be relevant to better understand the interdependence of tumors and the immune system in humans.

## Supplementary information

Supplemental Material
