# Supplementary material for: Enhanced differentiation of functional human T cells in NSGW41 mice with tissue-specific expression of human interleukin-7
Source: Leukemia. 2021 May 11;35(12):3561–7. doi: 10.1038/s41375-021-01259-5 (PMC8632686; doi:10.1038/s41375-021-01259-5)
Supplement: Supplementary file 1 — Supplemental Material [file 41375_2021_1259_MOESM1_ESM.pdf]

Supplemental Material

Enhanced differentiation of functional human T cells in NSGW41 mice with tissue-specific expression of human interleukin-7.

Emilie Coppin[1,2*], Bala Sai Sundarasetty[3*§], Susann Rahmig[1,2], Jonas Blume[4], Nikita A. Verheyden[3], Franz Bahlmann[5], Sarina Ravens[4], Undine Schubert[9,10], Janine Schmid[9,10], Stefan Ludwig[11], Katharina Geissler[12], Orlando Guntinas-Lichius[12], Constantin von Kaisenberg[6], Tanja Groten[13], Alexander Platz[7], Ronald Naumann[8], Barbara Ludwig[9,10], Immo Prinz[4], Claudia Waskow[1,2,9,14$], Andreas Krueger[3$]

Supplementary Materials and Methods

Characterization of BAC transgenic mice BAC integrity upon integration and copy numbers were determined by PCR. To this end, genomic DNA was isolated from transgenic mice and analyzed by SYBR green qPCR in a standard curve experiment to confirm close to 100% amplification efficiency. Amplification of hIL7 was normalized to the mouse TBP gene. The following primers were used: hIL7_fwd: CGCCAGAAAGCTGAGACAGT; hIL7_rev: GGAAGCACAGGTCGTTCAGT; TBP_gDNA_fwd GGCACAGGACTTACTCCACA; TBP_gDNA_rev: GGTGCAGTGGTCAGAGTTTG. mRNA expression of hIL-7 was analyzed by SYBR green qRT-PCR using the following primers: fwd: TCCTGATGGGCACCAAAG; rev: ATGGCTGGCAACTAGAAG. Offspring showing detectable expression of hIL-7 mRNA was crossed with NSGW41 mice [1] to generate the NSGW41hIL7 strain. Genotyping for the hIL-7 transgene was performed using the following primers: Tg-hIL7_fwd: ACGTGTCCTTCCGGTACATC; Tg-hIL7_rev: CTTCAGGAAGCACAGGTCGT. Detection of human IL-7: Human IL-7 from non-humanized and 28 to 40 weeks-old humanized mice (24 to 32 weeks after humanization) was quantified in serum, bone marrow and thymus supernatant (each 300µL) using the human IL7 procarta-plex kit (Thermofisher).

Human HSPC transplantation Cord blood samples were obtained from the Department Obstetrics, Gynecology and Reproductive Medicine, Hannover Medical School, Hannover, from the Bürgerhospital Frankfurt am Main, from the DKMS Cord Blood

Bank, Dresden, and from the Department of Obstetrics, Jena University Hospital, and were used in accordance with the guidelines approved by the ethics committees of Hannover Medical School, Frankfurt University Clinics, Dresden University of Technology, and University Clinics Jena. $CD34^+$ HSPCs were isolated using dual magnetic beads enrichment according to the manufacturer's instructions (Miltenyi Biotech) [1]. Purities >80% were considered acceptable. Contaminating T cell frequencies were routinely below 1%. Five – 10 * 10^4 HSPCs were transplanted intravenously into non-irradiated 4 to 10 weeks old male and female NSGW41 and NSGW41hIL7 mice. All mice with a blood percentage human $CD45^+$ cells >10% of total h+$mCD45^+$ cells 12 weeks post humanization were included in the analysis and no further selection was applied. All experiments were performed in order to determine the biological effects of human IL7 as a transgene in the NSGW41hIL7 mouse model and consequently and did not require any blinding. Sample sizes and groups were determined by the quantity of cord blood human $CD34^+$ HSPCs and the availability of age matched NSGW41 and NSGW41hIL7 recipients at humanization time points. Experimental groups for further analysis were then determined according to the available number of NSGW41 and NSGW41hIL7 recipients humanized at given experimental time points post human HSPC transplantation, with a minimum of at least 2 mice per group for each individual experiment. No statistical methods were used to determine sample size. No randomization of the groups was performed.

Flow cytometry Analysis was performed as described before [1]. A full list of antibody panels is provided in Table S1.

TCR repertoire analysis After mRNA isolation (Qiagen Micro Kit), cDNA was generated via the Smarter 5'RACE cDNA amplification kit (Clontech) using 4.5µL mRNA input and following the recommended protocol. Complementarity-determining region 3 (CDR3) regions of the human TRB locus were amplified through a gene-specific primer (2µM final concentration) that targets the constant region of the beta (β)-chain (GCACACCAGTGTGGCCTTTTGGG) and a primer (1µM final concentration) binding to the introduced SMARTER oligonucleotide (CTAATACGACTCACTATAGGGC) using the Advantage 2 PCR kit (Clontech) in a 50µL reaction. Both primer sequences further contain 16S Illumina overhang adapter sequences. Cycling conditions were as following: 120s 95°C; 30 times 30s 95°C, 45s 64°C, 60s 72°C; 60s 72°C. Generated PCR amplicons were agarose gel purified (Qiagen GelExtract.) Next, PCR samples were indexed with Nextera Illumina Indices reads using the Advantage 2 PCR kit

(Clontech) in a 8 PCR cycle reaction and purified with Agencourt AMPpure XP beads (Beckman Coulter) according to the manufacturers protocol. Samples were pooled, denatured and subjected to Illumina MiSeq analysis using 500 cycles and paired-end sequencing following Illumina guidelines. Sequencing libraries contained 20% PhIX for library complexity. Demultiplexed Fastq files were annotated to the human TRB locus via MiXCR software [2]. Individual CDR3 nucleotide sequences were ranked according to their abundance within the respective samples and further analyzed using VDJTools [3] and TcR [4]. TCR repertoire data are available at SRA (https://www.ncbi.nlm.nih.gov/sra), accession number PRJNA606460.

Islet xenotransplantation 500 IEQ (Islets Equivalent) adult pig islets or PBS were transplanted in the portal vein of NSGW41hIL7 mice that were humanized 24 weeks before. Islets were obtained from Goettingen minipigs (Ellgard) as described before [5,6]. Human blood cell chimerism of used mice was 52-85%. Regulatory T cells were analyzed 18 hours after surgery.

Detection of human immunoglobulins Human IgG1, IgG2, IgG3, IgG4, IgM, IgA and IgE were quantified in serum of humans and of humanized mice 26 to 32 weeks post humanization using the human antibody isotyping panel 7plex procarta-plex kit (Thermofisher).

T cell co-stimulation $hCD3^+$ T cells were isolated from the spleen of humanized NSGW41 or NSGW41hIL7 mice 16 to 25 weeks after HSPC transplantation, enriched using negative depletion (Miltenyi), and labeled with cell proliferation dye CPD (eBioscience). 10^5 $hCD3^+$ T cells were mixed with human T-Activator CD3/CD28 Dynabeads (Thermofisher, ratio 3:1) or PHA (1µg/mL) in RPMI 10% FCS, 20mM L-glutamine, 10mM Hepes, 1mM Sodium Pyruvate, 50µM ß-mercaptoethanol with recombinant hIL-2 (30 U/mL) and incubated for 6 days (37°C, 5% CO_2).

Immunofluorescence Mesenteric lymph nodes from 30 weeks humanized mice and human cervical lymph node were embedded in OCT and frozen. 10µm sections were fixed with 4% PFA, incubated 30min with PBS containing 10% normal rat serum (Abcam), Streptavidin and Biotin sites were saturated using the Streptavidin/Biotin blocking Kit (Vector Laboratories) according to the manufacturer's protocol, and stained with the antibodies and secondary reagents described in Table S1. Samples were analyzed using a fluorescence microscope (BL-9000E: Keyence) and images were processed using the BZ-Analyzer II (Keyence) and Adobe Photoshop CC 2019 softwares.

Statistics As data distribution is assumed to follow a normal distribution, one tailed Student's t tests were performed for all statistical analyses using Prism 8 for MacOSX software, allowing a significance level (alpha) of 0.05. In all graphs *p = 0.05-0.01, **p = 0.01-0.001, ***p = 0.001-0.0001 and ****p < 0.0001. Boxes and whisker plots display each individual data point as one dot and show data distribution in quartiles (boxes), the overall range (whiskers) and the median of the values. The data displayed as histograms in Fig. 1g, 2a and S2b represent the mean ± SD.

Table S1

Antibodies	Clone	Provider	Catalog #
hCD3 APC	UCHT1	eBioscience	17-0037-42
hCD3 AF750	UCHT1	eBioscience	27-0038-71
hCD3 APC Cy7	HIT3a	eBioscience	47-0038
hCD3 biotin	UCHT1	BioLegend	300404
hCD4 eF450	OKT-4	eBioscience	48-0048
hCD4 PE Cy7	RPA-T4	eBioscience	25-0049
hCD8 PE Cy5	RPA-T8	eBioscience	15-0088-42
hCD8 PerCP	SK1	eBioscience	9043-0087-025
hCD8 PE Cy7	RPA-T8	eBioscience	25-0088-41
hCD10 APC Cy7	HI10a	BioLegend	312212
hCD14 AF700	M5E2	BD Pharmingen	557923
hCD16 PerCP	3G8	BioLegend	302030
hCD16 PE Cy5	3G8	BD Biosciences	555408
hCD19 APC	HIB19	eBioscience	17-0199-42
hCD19 PC7	HIB19	eBioscience	25-0199-41
hCD19 FITC	HIB19	eBioscience	11-0199-73
hCD20 AF647	2H7	BioLegend	302318
hCD25 PE	M-A251	BD Biosciences	555432
hCD31 PE	MBC782	BD Pharmingen	566125
hCD33 PE	WM-53	eBioscience	12-0339-42
hCD33 PE Cy7	WM-53	eBioscience	25-0338-42
mCD45 APC eF780	30F11	eBioscience	47-0451-82
mCD45 AF700	30F11	eBioscience	56-0451-82
hCD45 eF450	HI30	eBioscience	48-9998-41
hCD45 FITC	HI30	eBioscience	11-0459
hCD45 PE Cy7	HI30	BioLegend	304015
hCD45RA eF450	HI100	eBioscience	45-0458
hCD45RO FITC	UCHL1	BD Biosciences	555492
hCD62L Biotin	DREG.55	eBioscience	46-0626
hCD69 PerCP eF710	FN50	BioLegend	310926
hCD235 FITC	GA-R2 (HIR2)	eBioscience	11-9987-82
hFoxp3 APC	PCH101	eBioscience	17-4776-42
hHLA DR PerCP Cy5.5	4S.B3	BioLegend	307629
hIgD PE	IA6-2	BD Pharmingen	555779
hIgM Biotin	SA-DA4	BioLegend	314504
mTer119 FITC	Ter119	eBioscience	11-5921-82
anti-FITC AF488	n/a	Thermofisher	A11096
streptavidin V500	n/a	BD Biosciences	561419
streptavidin FITC	n/a	BD Pharmingen	554020
streptavidin Cy3	n/a	Thermofisher	016-160-054
Cell Proliferation Dye eF450	n/a	ebioscience	65-0842-85
Fixable viability dye AF700	n/a	BD Biosciences	564997

Supplementary figures

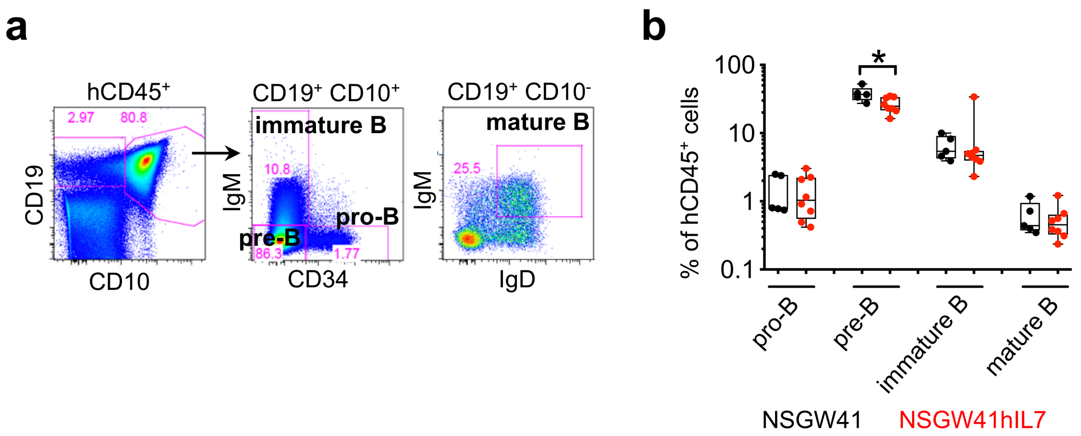

Supplementary Figure 1: B lymphopoiesis in NSGW41hIL7 mice. (**a**) Dot plots show the gating strategy of bone marrow human B lineage subsets: pro-B (hCD45$^+$ CD19$^+$ CD10$^+$ IgM$^-$ CD34$^+$), pre-B (hCD45$^+$ CD19$^+$ CD10$^+$ IgM$^-$ CD34$^-$), immature B (hCD45$^+$ CD19$^+$ CD10$^+$ IgM$^+$ CD34$^-$) and mature B cells (hCD45$^+$ CD19$^+$ CD10$^-$ IgM$^+$ IgD$^+$). (**b**) Frequencies of B cell subsets in bone marrow of humanized mice 26 weeks after humanization. Data are pooled from 2 independent experiments.

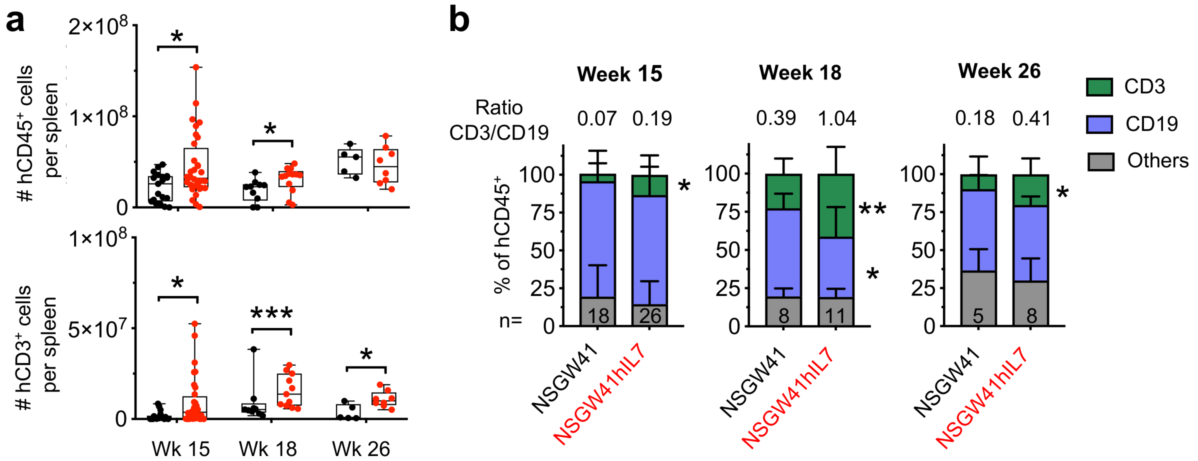

Supplementary Figure 2: Human lymphocyte composition in spleen. (**a**) Numbers of $hCD45^+$ leukocytes (top) and $hCD3^+$ T cells (bottom) in spleens of NSGW41 or NSGW41hIL7 mice 15, 18 and 26 weeks after humanization. (**b**) Frequencies of $hCD3^+$, $hCD19^+$, and other cells within $hCD45^+$ leukocytes in spleens of NSGW41 or NSGW41hIL7 mice 15, 18 and 26 weeks after humanization. Numbers on top of graphs indicate T vs. B cell ratio. Numbers in the bottom of each column indicate the number of mice per group. Data are pooled from at least 2 independent experiments.

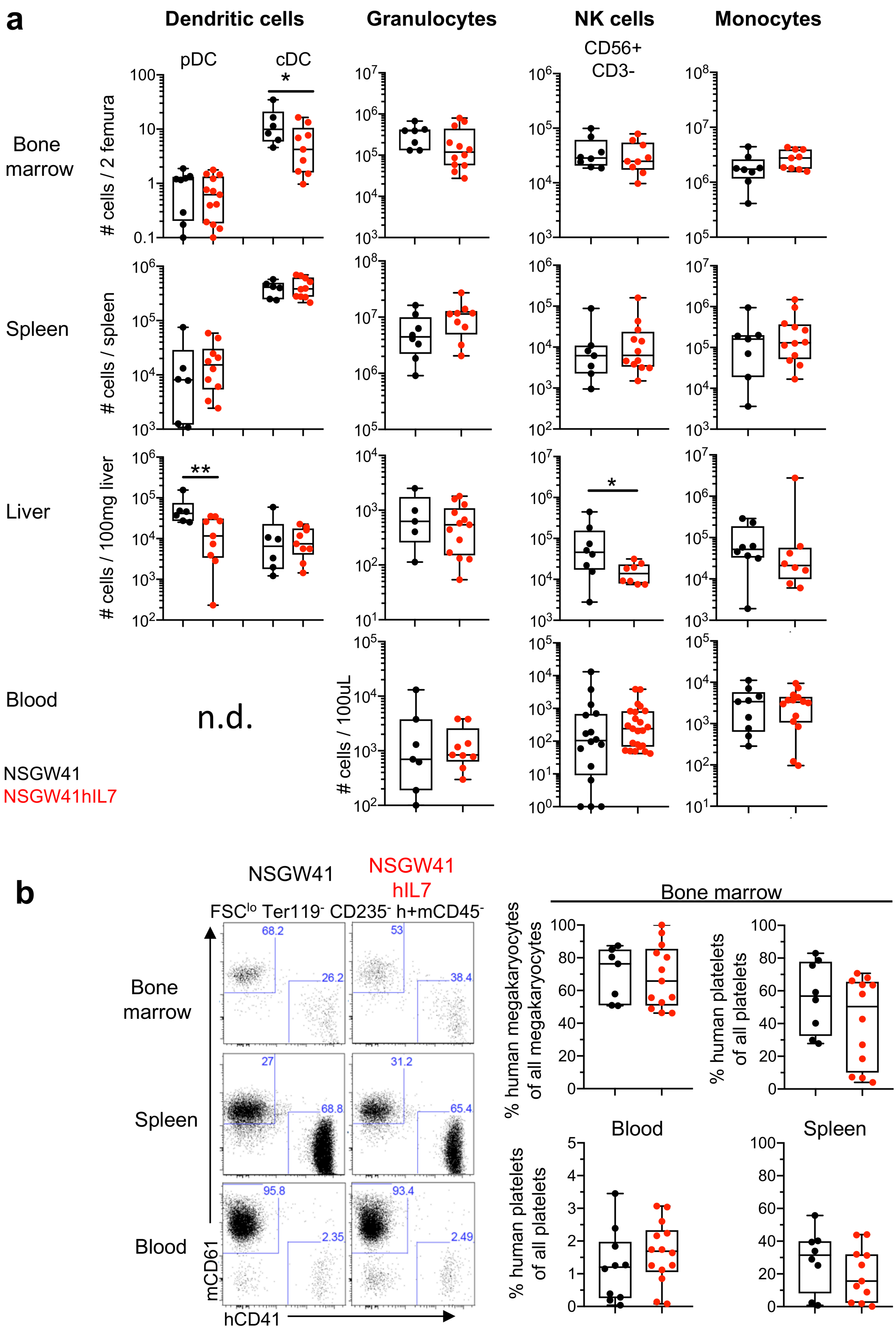
a
Dendritic cells
Granulocytes
NK cells
Monocytes
pDC
cDC
CD56+
CD3-
Bone marrow
Spleen
Liver
Blood
cells / 2 femura
cells / spleen
cells / 100mg liver
cells / 100uL
n.d.
NSGW41
NSGW41hIL7
b
NSGW41
NSGW41 hIL7
FSClo Ter119- CD235- h+mCD45-
mCD61
hCD41
68.2
26.2
53
38.4
27
68.8
31.2
65.4
95.8
2.35
93.4
2.49
Bone marrow
% human megakaryocytes of all megakaryocytes
% human platelets of all platelets
Blood
Spleen

Supplementary Figure 3: Numbers of myeloid cells, NK cells, megakaryocytes and platelets in NSGW41 and NSGW41hIL7 mice. (**a**) Bone marrow, spleen, liver and blood from humanized NSGW41 or NSGW41hIL7 mice were analyzed 26-32 weeks after humanization. (Plasmacytoid DCs $hCD45^{+}$ $CD123^{+}$ $CD11c^{-}$; cDCs $hCD45^{+}$ $CD11c^{+}$ $CD123^{-}$; granulocytes, $hCD45^{+}SSC^{hi}$; NK cells, $hCD45^{+}$ $CD33^{-}$ $CD19^{-}$ $CD3^{-}$ $CD56^{+}$; monocytes $hCD45^{+}$ $CD33^{+}$ SSC^{int}. (**b**) Left: Dot plots resolve FSC^{lo} $Ter119^{-}$ $CD235^{-}$(h+m)$CD45^{-}$ cells for the expression of mouse ($mCD61^{+}$) or human ($hCD41^{+}$) surface markers. Right: Frequencies of human megakaryocytes (FSC^{hi} $Ter119^{-}$ $CD235^{-}$ (h+m)$CD45^{-}$ $hCD41^{+}$) among total megakaryocytes in bone marrow and human platelets among total platelets in bone marrow, spleen and liver. Each dot represents an individual mouse. Data were pooled from 2 independent experiments.

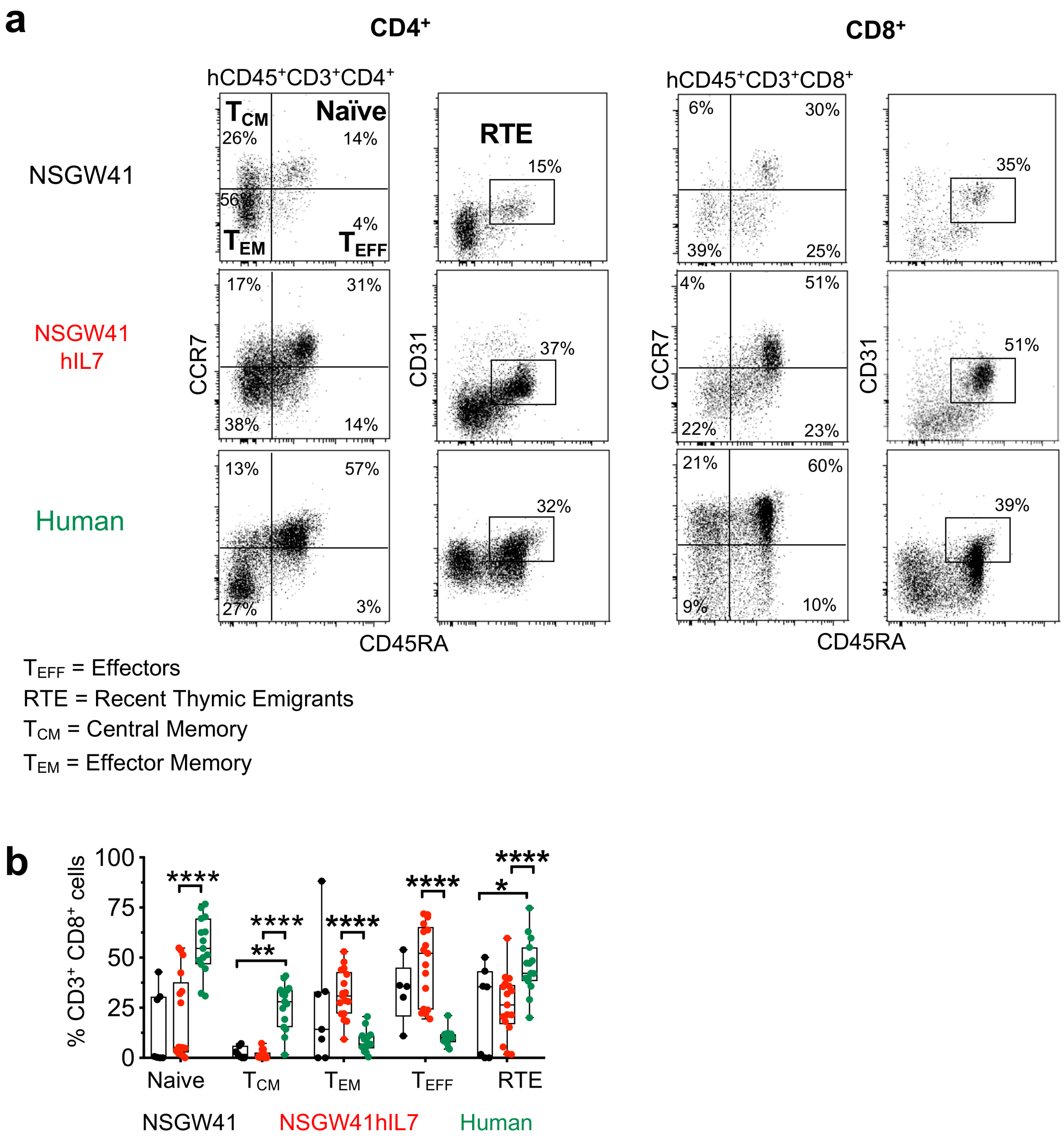

Supplementary Figure 4: Characterization of human T cell subpopulations in the blood. (**a**) Gating strategy and identification of $hCD4^+$ and $hCD8^+$ T cell subpopulations in blood of humanized mice: T effectors (T_{EFF}, $hCD45^+$ $CD3^+$ $CD4^+$ $CCR7^-$ $CD45RA^+$), central memory (T_{CM}, $hCD45^+$ $CD3^+$ $CD4^+$ $CCR7^+$ $CD45RA^-$), effector memory (T_{EM}, $hCD45^+$ $CD3^+$ $CD4^+$ $CCR7^-$ $CD45RA^-$), naïve T (Naïve, $hCD45^+$ $CD3^+$ $CD4^+$ $CCR7^+$ $CD45RA^+$) and recent thymic emigrants (RTE, $hCD45^+$ $CD3^+$ $CD4^+$ $CD31^+$ $CD45RA^+$). (**b**) Composition of blood $CD8^+$ T cell subpopulations 26 weeks after humanization. Data was acquired 26 weeks after humanization. The composition of subpopulations in human blood is shown for comparison (bottom). Data were pooled from 2 independent experiments.

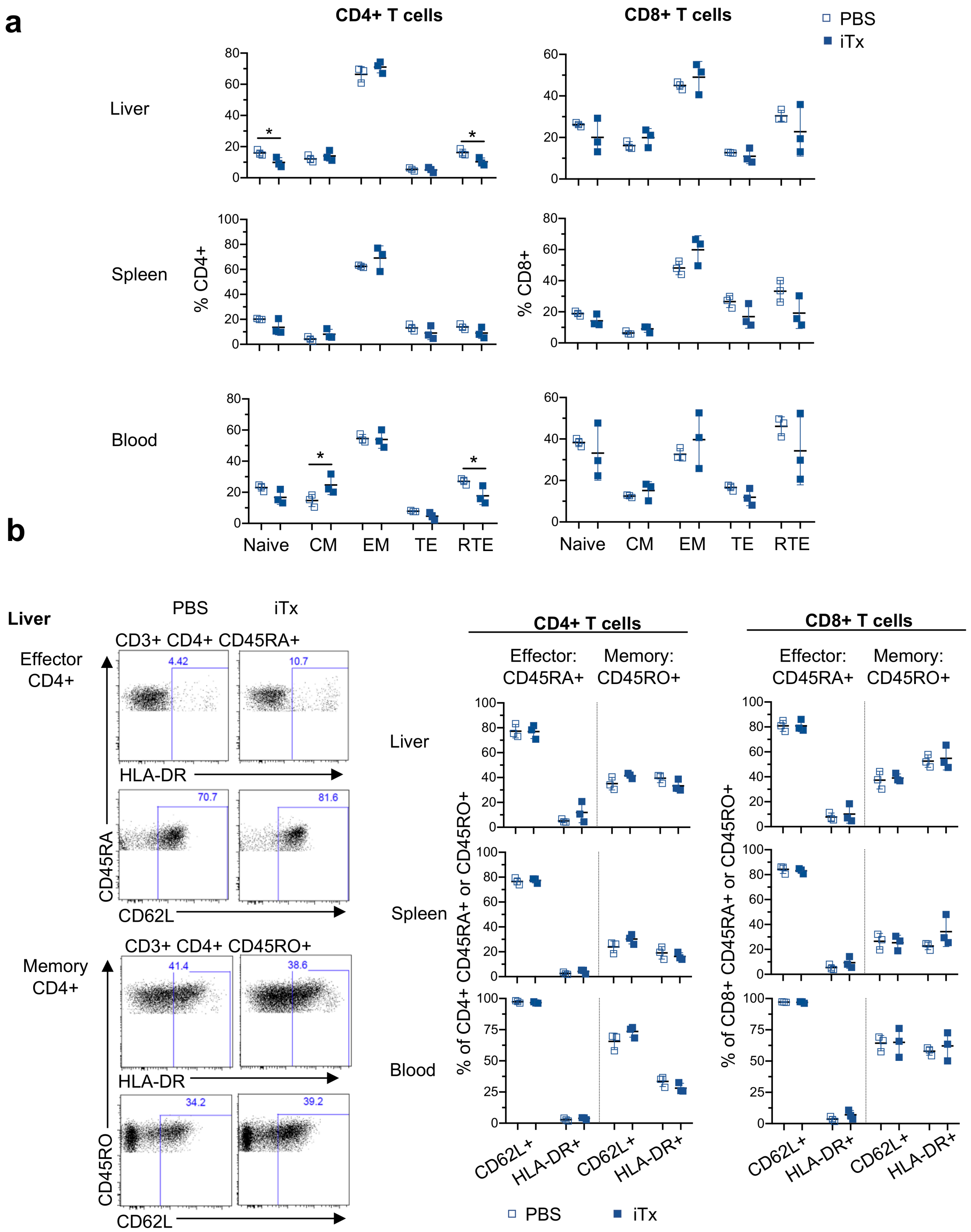

Supplementary Figure 5: Characterization of human T cell subpopulations after islet xenotransplantation in liver, spleen, and blood. (**a**) Composition of T cell subpopulations identified as shown in Supplementary Figure 4. (**b**) Left: representative FACS plots showing expression of HLA-DR and CD62L on effector T cells (CD45RA^{+}, top) and memory T cells (CD45RO^{+}, bottom) in the liver. Right: Quantitative analysis of activation of hCD4^{+} and hCD8^{+} T cells upon islet xenotransplantation (iTx) in the liver, spleen and blood. n=3 in each group, data representative of 1 experiment.

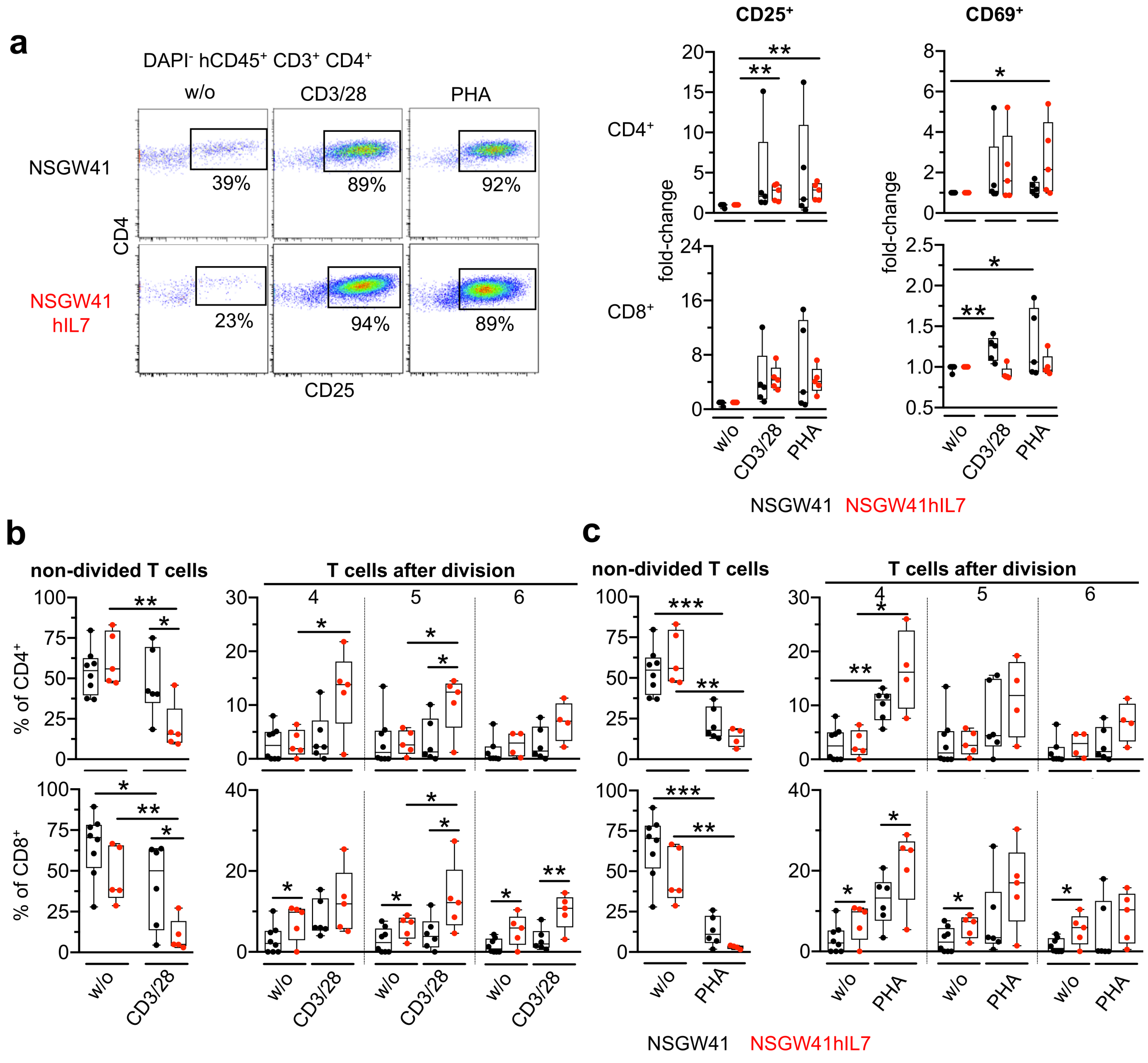

Supplementary Figure 6: Activation of human T cells from NSGW41 or NSGW41hIL7 mice. (**a**) Dot plots show the expression of CD25 and CD69 on hCD4^{+} T cells isolated from NSGW41 (top) or NSGW41hIL7 (bottom) spleens after 6 days of CD3/28 or PHA stimulation or non-stimulated (w/o) controls. Right: fold-changes were calculated by dividing the percentages of stimulated CD25^{+} or CD69^{+} hCD4^{+} (top) or hCD8^{+} (bottom) splenic T cells by the percentages of non-stimulated cells (w/o) from NSGW41 or NSGW41hIL7 mice. (**b**) Frequencies of non-divided human T cells and T cells that have divided 4, 5 or 6 times 6 days after stimulation with CD3/28 antibodies or controls (right). Top: CD4^{+} T cells, bottom: CD8^{+} T cells. (**c**) Percentages of non-divided T cells and T cells which have undergone 4, 5 or 6 divisions 6 days after stimulation with phytohemagglutinin (PHA) or without stimulation (w/o). Top: hCD4^{+} T cells, bottom: hCD8^{+} T cells. Donor mice had received human HSPCs 20 to 26 weeks before. Data are pooled from 2 independent experiments.

Supplemental References

1. Cosgun KN, Rahmig S, Mende N, et al. Kit Regulates HSC Engraftment across the Human-Mouse Species Barrier. *Cell Stem Cell*. 2014;15(2):227-238.
2. Bolotin DA, Poslavsky S, Mitrophanov I, et al. MiXCR: software for comprehensive adaptive immunity profiling. *Nat Methods*. 2015;12(5):380-381.
3. Shugay M, Bagaev DV, Turchaninova MA, et al. VDJtools: Unifying Post-analysis of T Cell Receptor Repertoires. *PLoS Comput Biol*. 2015;11(11):e1004503.
4. Nazarov VI, Pogorelyy MV, Komech EA, et al. tcR: an R package for T cell receptor repertoire advanced data analysis. *BMC Bioinformatics*. 2015;16:175.
5. Ludwig B, Ludwig S, Steffen A, et al. Favorable outcome of experimental islet xenotransplantation without immunosuppression in a nonhuman primate model of diabetes. *Proc Natl Acad Sci U S A*. 2017;114(44):11745-11750.
6. Steffen A, Kiss T, Schmid J, et al. Production of high-quality islets from goettingen minipigs: Choice of organ preservation solution, donor pool, and optimal cold ischemia time. *Xenotransplantation*. 2017;24(1).
